# Utilization Patterns of Hospital Biobank Samples: Time to First Use and Material-Specific Demand

**DOI:** 10.3390/jcm15114292

**Published:** 2026-06-01

**Authors:** Patrick Mucher, Manuela Bayer, Ines Flieder, Gabriela T. Humer, Lilian Hou, Adelheid Koll, Astrid Radakovics, Natasa Ristic, Thomas Schickbauer, Wilfried Müller, Van-Lin Nguyen, Michael Kim-Tran, Alexander Szabo, Johannes A. Hainfellner, Philipp Hofer, Thomas Perkmann, Oswald F. Wagner, Helmuth Haslacher

**Affiliations:** 1 Department of Laboratory Medicine, Medical University of Vienna, 1090 Vienna, Austria; patrick.mucher@meduniwien.ac.at (P.M.);; 2 Division of Neuropathology and Neurochemistry, Department of Neurology, Medical University of Vienna, 1090 Vienna, Austria; 3 Department of Pathology, Medical University of Vienna, 1090 Vienna, Austria

**Keywords:** biobank utilization, sample management, storage time, aliquot patterns, prospective biobanking

## Abstract

**Objectives**: Biobanks collect biological material prospectively for future, yet unspecified research needs. The relationship between collection strategy and actual utilization remains poorly documented, yet understanding usage patterns is critical for resource allocation and planning. We aimed to analyze the time until first use and the utilization rates stratified by collection type and sample material. **Methods**: We conducted a retrospective analysis of metadata for all submissions (patient sample set per timepoint) deposited in 2019 (*n* = 13,666 from 56 collections) at the MedUni Wien Biobank. Collections were classified as prospective cohorts (without predefined hypotheses) or dedicated studies. Utilization was assessed at three levels: collections accessed, submissions from which at least one aliquot was requested, and total aliquots distributed. **Results**: Within 6–7 years, 31/56 collections (55%) were accessed, with 3466/13,666 submissions (25.4%) and 7565 of ~218,000 aliquots (3.5%) utilized. Among accessed collections, 21/35 were prospective cohorts, and 10/21 were dedicated studies. Median submission utilization was 54.2% (Q1–Q3: 25.2–93.1%). Among accessed submissions, conditional median time was 926 days (Q1–Q3: 438–1573). Serum was most frequently requested (24/31 projects). Aliquot requests per submission varied by material: median of 1 aliquot per sample for all materials except for citrate (2) and lithium heparin (5) plasma. Four material types showed no demand: buffy coat, ccfDNA, double-centrifuged citrate plasma, and saliva. **Conclusions**: The 3.5% aliquot-level utilization rate, numerically below internationally reported 10–20%, reflects metric granularity and the broad aliquoting strategy rather than low sample demand. Material-specific timing and demand patterns provide an evidence base for resource planning in hospital-based biobanks.

## 1. Introduction

Biobanks, which are facilities responsible for the acquisition, processing, storage, and distribution of biomaterials and associated data, have emerged as indispensable research infrastructures over recent decades [1]. On the one hand, biobanks facilitate more efficient research workflows by providing centralized sample logistics, allowing individual research groups to focus on hypothesis generation and testing. On the other hand, these facilities aim to increase research reliability by applying validated processing methods [2] and contributing their expertise regarding sample fitness for purpose, thereby addressing the problem of non-reproducibility [3,4,5].

When new biobanks are established, management must strike a balance between sampling strategy (e.g., how narrowly inclusion criteria are defined, how many different materials are collected per sampling event, how many aliquots are generated from each primary sample) and resource consumption. Broader sampling strategies enable a wider range of research questions to be addressed, but require more storage space, equipment, and personnel, directly affecting the biobank’s sustainability [6,7,8]. In addition, and increasingly important in the context of the ongoing climate crisis, they demand more energy, thereby increasing the biobank’s carbon footprint. Recent surveys suggest that typical biobank utilization rates range from 10 to 20%, with many biobanks reporting target utilization rates 2.5 to 5 times higher than achieved rates [9,10]. However, detailed analyses of time from storage to first use, material-specific utilization patterns, and differences between collection strategies are largely absent from the literature.

However, there is still limited data on the extent to which distinct biobank samples are utilized, although such information is highly warranted for biobanks that must decide how many samples to store and what level of access to expect. Understanding temporal patterns of sample utilization has direct implications for biobank sustainability, optimizing collection protocols, and strategic planning. Knowledge of material-specific usage patterns can inform the planning of aliquot numbers, storage space allocation, and cost calculations. Furthermore, empirical data on utilization timing can support evidence-based approaches to biobank quality management and performance assessment.

Filling this gap with data from a large Austrian hospital-based biobank (MedUni Wien Biobank, www.biobank.at), the present study aimed to analyze time to first use of biobanked samples stratified by collection type and biological material, to characterize utilization rates across different collection approaches, to identify material-specific patterns in both timing and quantity of sample requests, and to provide benchmark data for hospital-based biobanking operations.

## 2. Materials and Methods

### 2.1. Study Design

This retrospective analysis included all submissions received in 2019. The year 2019 was chosen as it preceded the COVID-19 pandemic, avoiding potential confounding effects of pandemic-related disruptions to biobank operations and research activities. Follow-up extended until December 2025.

Storage and access data were extracted from the local information and management systems, including submission date, collection identifier, sample material type, date of first request, and number of aliquots. 

### 2.2. Definitions

A “submission” is defined as a complete set of biological samples collected from one patient at one time point. Each submission may include multiple sample types and multiple aliquots per sample type. A “collection” is a defined set of submissions collected according to a standard protocol and usually governed by a single ethics approval. Collections were classified as “prospective cohorts” (broad collection without predefined specific hypotheses) or “dedicated studies” (collection linked to particular research projects) based on information in the biobank’s collection registry. For prospective cohorts, sample utilization is by definition not pre-planned at the time of collection. For dedicated studies, utilization is typically anticipated within the study protocol but not guaranteed; moreover, standard practice is to store more aliquots than required for the planned analyses, resulting in residual samples that may or may not be accessed subsequently. “Time to first use” is defined as the interval in days between the submission storage date and the date of the first aliquot request from that submission. “Submitters” are clinical departments that contribute samples to the biobank, while “users” are researchers who request samples; however, in most cases, submitters are also the primary users of the samples.

### 2.3. Biobank Description

The MedUni Wien Biobank is a joint venture among three diagnostic units of the Medical University of Vienna (the Department of Laboratory Medicine, the Department of Pathology, and the Division of Neuropathology and Neurochemistry of the Department of Neurology). Most liquid collections are processed, stored, and distributed at the Department of Laboratory Medicine within Austria’s largest university hospital. The biobank operates within a quality management system certified to ISO 9001:2015 and implements technical specifications for pre-examination processes, including CEN/TC 140 and ISO/TC 212 standards and specifications (see BBMRI-ERIC directory). The facility participates in the Austrian biobank consortium BBMRI.at, which serves as the national node of BBMRI-ERIC, the European biobanking research infrastructure (https://www.biobank.at, accessed on 1 April 2026).

Sample collection, processing, and storage follow standardized protocols integrated into routine clinical workflows. Samples are transported to the biobank facility via a pneumatic tube system or courier service, where they undergo centrifugation, aliquoting, and cryopreservation according to collection-specific protocols. Long-term storage occurs at −80 °C in automated freezers with continuous temperature monitoring and alarm systems. Each submission (defined as the complete set of samples from one patient at one timepoint) is registered in the laboratory information management system (MOLIS 40 U10.4, Sysmex, Kobe, Japanv), along with associated metadata including the collection identifier, sample types, processing parameters, and storage locations.

Sample requests are formally initiated by contacting the biobank via the BBMRI-ERIC network or dedicated channels (e.g., access-biobank@meduniwien.ac.at) and follow one of three standardized access pathways (cf. https://www.meduniwien.ac.at/web/en/biobank/allgemeine-information/information-fuer-forscherinnen/probenzugriff/, accessed on 1 April 2026): (a) sample retrieval as part of an ethics-approved project that is itself linked to an associated biobank collection, requiring the request to specify project number, required material type, and volume; (b) sample retrieval as part of an ethics-approved project that is not itself an independent biobank, in which case sample availability is first assessed and approval is then granted in coordination with the responsible project management; and (c) an advisory service in which researchers without a pre-existing collection submit their scientific objectives, intended analyses, required material type, volume, and disease focus, and the biobank performs a sample search and facilitates contact with the sample contributor. Of note, samples deposited in the MedUni Wien Biobank are usually collected under broad informed consent that allows their re-use in subsequent biomedical research projects beyond the original study purpose. Each individual downstream use, however, must still be covered by a specific ethics vote (or amendment) by the Ethics Committee. Donors retain the right to revoke their consent at any time, in which case all remaining samples and associated data are destroyed; until such revocation, samples may be used within the scope of approved projects. Quality certifications held by the biobank (ISO 9001:2015 quality management; compliance with CEN/TS and ISO/TS pre-examination standards) are listed in the BBMRI-ERIC directory and are routinely communicated to potential users on request. In practice, however, the majority of samples are accessed via the clinical partner who originally collected them—either because the clinician initiates the analysis themselves, or because they enter into a cooperation with external researchers, since the clinical partner is also the gatekeeper of the linked clinical data. The biobank therefore serves as a networking platform that connects interested external researchers with relevant clinical contributors when access to clinical annotations is required.

### 2.4. Stored Materials Within the Observation Period

Collected materials included i.a. serum, citrate plasma, lithium heparin plasma, EDTA plasma, DNA/whole blood, urine, faeces, cerebrospinal fluid, buffy coat, double-spun plasma for future isolation of cell-free circulating DNA, double-centrifuged citrate plasma, cyst punctates, PAXgene RNA tubes (PreAnalytiX GmbH, Hombrechtikon, Switzerland), and saliva. Most blood- or urine collection tubes were produced by Greiner Bio-One GmbH, Kremsmuenster, Austria.

Aliquot volumes are collection-specific, but they are typically 400 µL for urine, CSF, serum, and plasma, except for citrate plasma (i.e., 300 µL) and double-spun plasma for ccfDNA isolation (2 mL). DNA is stored in aliquots of 100 µL, and whole blood in primary tubes (3 mL). Faeces is stored either native or as swabs in primary tubes or in aliquots of 2 mL.

All materials, except whole blood to some extend urine or stool, were processed either manually or using Roche cobas RSD/RSA pre-analytical systems (Roche Diagnostics, Rotkreuz Switzerland), aliquoted into 2D-barcoded tubes (mainly LVL technologies GmbH & Co. KG, Crailshaim, Germany or Thermo Fisher Scientific, Waltham, MA, USA), and stored at <−70 °C according to standard operating procedures. Whole blood was stored in the primary container for most collections at −20 °C or below. SPRECs (Standard PREanalytical Code, v2.0) [11] can be derived from Table 1.

### 2.5. Statistical Analysis

Descriptive statistics included medians with first and third quartiles (Q1, Q3) and ranges for continuous variables, and frequencies with percentages for categorical variables. Time to first use was analyzed using Kaplan–Meier survival analysis including all submissions, with first access as event and end of follow-up (31 December 2025) as censoring date for submissions not accessed; differences between material types were compared using the log-rank test. Conditional descriptive statistics (median, IQR, range) were calculated among accessed submissions only. Likewise, material-specific aliquot requirements were analyzed by calculating median, mean, and range of aliquots requested per submission for each material type, considering only submissions from which the respective material was actually requested. Utilization was calculated at three levels: collections accessed relative to total collections, submissions accessed relative to total submissions, and aliquots distributed relative to total stored aliquots. Comparisons between collection types (prospective cohorts vs. dedicated studies) were accomplished by Mann–Whitney U tests or Pearson’s χ^2^-tests. A two-sided *p*-value < 0.05 was considered statistically significant. Statistical analyses were conducted using MedCalc v23.5.2 (MedCalc, Ostend, Belgium) and GraphPad Prism v11 (GraphPad, La Jolla, CA, USA).

## 3. Results

### 3.1. Utilization Overview

During 2019, the MedUni Wien Biobank received 13,666 submissions distributed across 56 distinct collections. By the end of 2025, representing a maximum follow-up period of 6–7 years, 31 collections (55%) had supported research projects, with a total of 3466 submissions accessed (25.4% of all deposited submissions). In terms of single aliquots, a total of 7565 aliquots were distributed from approximately 218,000 stored aliquots, yielding an aliquot-level utilization rate of 3.5%.

**Figure 1:**
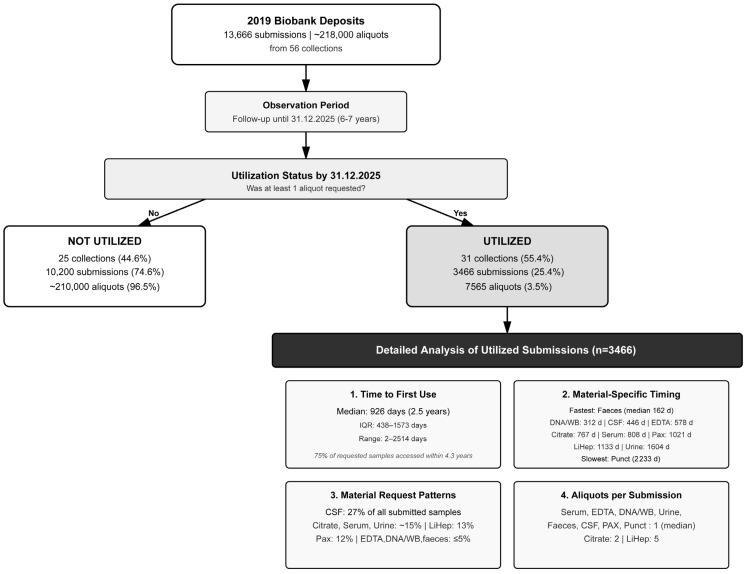
Study design and utilization outcomes. Of 13,666 submissions deposited in 2019, 3466 (25.4%) had at least one aliquot requested within the 6–7 year follow-up, illustrating how utilization rates differ markedly depending on whether collections, submissions, or aliquots are used as the denominator. LiHep, lithium heparin plasma; EDTA, EDTA plasma; DNA/WB, DNA or EDTA-anticoagulated whole blood for DNA isolation; CSF, cerebrospinal fluid; d, days.

Notably, the 31 utilized collections accounted for 93% (12,693) of all submissions during the observational period. A study flowchart is presented in Figure 1.

Of the 31 utilized collections, 21 were classified as prospective cohorts, while 10 were dedicated studies. Prospective cohorts demonstrated a numerically higher, yet statistically non-significant utilization rate (21/35, 60%) compared to dedicated studies (10/21, 48%, χ^2^ = 0.752, *p* = 0.386). Among the 31 collections accessed, the median submission utilization rate was 54.2% (IQR: 25.2–93.1%, range: 1.2–100%). This indicates that in a typical utilized collection, approximately half of all deposited submissions were requested for research use within 6–7 years. However, substantial heterogeneity existed across collections, with eleven achieving utilization rates above 90%, while two showed rates below 10%. Collection-specific metrics are provided in Table 2 and Table 3.

### 3.2. Material-Specific Request Patterns

Serum was the most frequently requested material, accessed from 24 of 31 collections (77%), followed by EDTA plasma (13 collections, 42%), citrate plasma (10 collections, 32%), DNA/whole blood (6 collections, 19%), and urine (5 collections, 16%). Cerebrospinal fluid, lithium heparin plasma, faeces, PAXgene RNA tubes, and punctates were each accessed from single collections. After the observation period, 26.5% of all submitted CSF samples, 15.3% of citrate plasma, 14.0% of serum, 14.1% of urine, 12.5% of lithium heparin plasma, 4.2% of EDTA plasma, 4.7% of faeces, 5.0% of DNA/EDTA whole blood samples, 11.5% of PAXgene RNA tubes, and all submitted punctates had been accessed.

Four material types that were prospectively collected showed no documented requests during the entire observation period: buffy coat, plasma containing cell-free circulating DNA (ccfDNA), double-centrifuged citrate plasma, and saliva.

### 3.3. Time to First Use

Kaplan–Meier analysis was performed including all submissions (with unused samples censored at end of follow-up) from 2019 and access times were stratified according to material type. Materials, for which less than 30 samples were collected, were summarized as “others” (i.e., umbilical chord serum, synovial fluid, saliva, buffy coat, cyst punctates and double-spun citrate plasma). However, as median times are defined as those times with 50% of the samples accessed, they could not be calculated for any material (access rates ranged from 0 to 21.4%). Log-rank test revealed significant differences in access patterns across material types (χ^2^ = 1202, *p* < 0.0001). CSF showed the highest overall access probability (restricted mean survival time [RMST] 2141 days), followed by serum (2323 days), citrate plasma (2328 days), and lithium heparin plasma (2359 days). EDTA plasma (2478 days), DNA/whole blood (2460 days), and faeces (2459 days) had longer access times. Notably, faeces exhibited the shortest conditional time to first use among accessed samples (median 162 days) but a low overall utilization rate (4.7%), illustrating that rapid access in individual cases does not necessarily translate to high cumulative utilization. Among accessed submissions, conditional median time from storage to first request was 926 days (IQR: 438–1573, range: 2–2514 days). Material-specific analysis revealed distinct patterns (Table 4).

**Figure 2:**
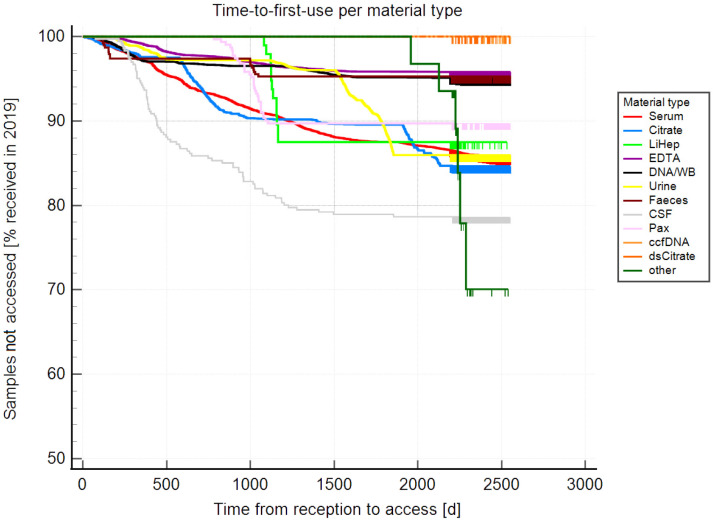
Kaplan–Meier curves for time to first use by material type. All submissions deposited in 2019 are included; those not accessed by 31 December 2025 are censored (tick marks). Curves show proportion of samples not yet accessed over time. No material reaches 50% cumulative utilization within the 6–7 year follow-up; curves diverge markedly, with serum showing a near-linear access pattern and most other materials showing stepwise (batchwise) accrual. Cit, citrate plasma; LiHep, lithium heparin plasma; EDTA, EDTA plasma; DNA/WB, DNA or whole blood; CSF, cerebrospinal fluid; Pax, PAXgene RNA; Punct, punctate.

Access curves (Figure 2) reveal material-specific request patterns. Whilst CSF and EDTA plasma are used mainly within the first three to four years of storage, serum seems to be continuously accessed, as indicated by the nearly linear curve. Lithium heparin plasma, citrate plasma, faeces, urine, and special materials, such as PAXgene RNA tubes or punctates, are, in contrast, accessed in batches at similar times after collection, corresponding to distinct research questions and utilizing a considerable share of the samples.

**Figure 3:**
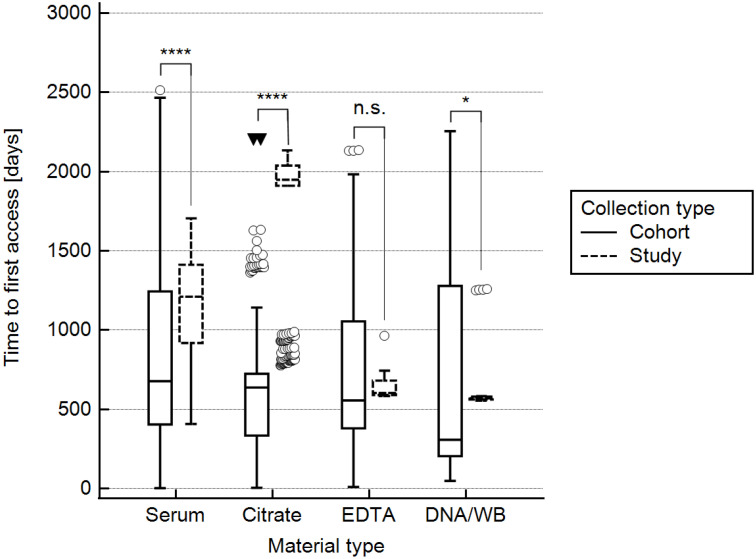
Time to first access stratified by material type (x-axis) and collection type in samples that were actually accessed. Access times were compared for each material type between samples collected in the framework of prospective cohorts or dedicated studies by Mann–Whitney U-tests. Samples from prospective cohorts were accessed significantly earlier than samples from dedicated studies for serum, citrate plasma, and DNA/whole blood, supporting the value of hypothesis-free collection strategies for rapid downstream use. EDTA, EDTA plasma; DNA/WB, DNA/whole blood; ****, *p* < 0.0001; *, *p* < 0.05; n.s., not significant.

Collection type significantly influenced access times in those samples that were actually accessed for serum, citrate plasma and DNA/whole blood, with prospective cohort samples accessed earlier than dedicated study samples (see Figure 3): for serum, median time was 677 days (IQR: 404–1244, range: 2–2514) for prospective cohort samples vs. 1211 (IQR: 918–1412, range: 407–1705) for dedicated study samples (*p* < 0.0001); for citrate plasma, 638 (IQR: 223–724, range: 4–2204) vs. 1949 (IQR: 1911–2038, range: 776–2134) days (*p* < 0.0001); for DNA/whole blood, 308 (IQR: 204–1279, range: 48–2255) vs. 570 (IQR: 562–580, range: 554–1257) days (*p* = 0.020). No significant differences were observed for EDTA plasma (*p* = 0.630). Other materials lacked sufficient data for comparison.

### 3.4. Aliquot Requirements

**Figure 4:**
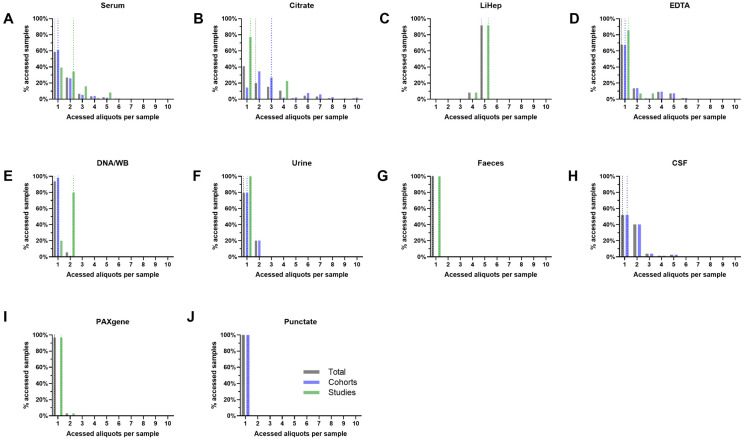
Distributed aliquots per accessed sample, stratified for material type (**A**–**J**). CSF, cerebrospinal fluid. Blue columns represent aliquots stored in the framework of prospective cohorts, green columns are aliquots derived from dedicated studies, and black columns are all accessed samples regardless of collection type. Vertical lines indicate median accessed aliquots per sample type (black: total; blue: cohorts; green: studies). Most material requests are satisfied by 1–2 aliquots per submission; only citrate plasma and lithium heparin plasma show substantially higher aliquot demand, suggesting that material-specific (rather than uniform) aliquoting could meaningfully reduce stored volumes without compromising research use.

The number of aliquots requested per submission varied substantially by material type (Table 5). Serum, EDTA plasma, DNA/whole blood, urine, faeces, CSF, PAXgene RNA, and punctates required a median of a single aliquot per submission. Larger volumes were required for citrate plasma (median = 2) and lithium heparin plasma (median = 5), with the latter requested only from a single collection. Mean values exceeded medians for most material types, indicating right-skewed distributions with some submissions requiring substantially more aliquots than typical. Detailed aliquot distributions are provided in Figure 4.

Collection type influenced aliquot requirements differentially across materials (Figure 4). Study samples required more aliquots than cohort samples for serum (2, IQR: 1–3, range: 1–6 study vs. 1, IQR: 1–2, range 1–9 cohort; *p* < 0.0001) and DNA/whole blood (2, IQR: 2–2, range: 1–2 study vs. 1, IQR 1–1, range: 1–4 cohort; *p* < 0.0001). Conversely, fewer aliquots from citrate plasma derived from studies were requested (1, IQR: 1–1, range: 1–4 study vs. 3, IQR: 2-3, range 1–10 cohort; *p* < 0.0001). No significant difference was observed for EDTA plasma (*p* = 0.121); insufficient data were available for the other materials.

## 4. Discussion

This retrospective analysis provides empirical data on temporal utilization patterns and material-specific requirements in a hospital-based biobank. Three principal findings emerged: first, utilization ranged from 3.5% at the aliquot level over 25.4 on the submission level to 55% at the collection level; second, Kaplan–Meier analysis revealed material-specific access patterns, with conditional median time to first use of 926 days among accessed submissions; and third, aliquot requirements showed distinct material-dependent patterns.

Overall, 55% of collections, 25% of all submissions and 3.5% of all stored aliquots were accessed within the 6–7 year follow-up period, a figure that fits with previously reported average utilization rates of 10–20% from international surveys of academic biobanks, depending on the comparison framework chosen—however, these figures are difficult to compare, as this metric is far from being standardized (e.g., whether accessed aliquots are compared to stored aliquots or if primary containers or submissions are used as a reference). For instance, the 3.5% aliquot-level utilization rate would fall below the 10–20% range reported in international surveys of academic biobanks [9,10], although this discrepancy likely reflects metric granularity rather than underperformance: the same data yield 25.4% at the submission level and 55% at the collection level, both within or above internationally reported ranges. This three-tiered comparison (3.5% vs. 25.4% vs. 55%) illustrates how profoundly the choice of denominator influences utilization metrics and underscores the need for standardized reporting.

The lower aliquot-level utilization rate raises questions about collection efficiency. Of 4537 material-level requests, 65% were satisfied by a single aliquot and 86% by two or fewer. Capping routine aliquoting at three per material type would have covered 91% of requests and 89% of demanded aliquots, while substantially reducing storage requirements. However, such caps would disproportionately affect citrate plasma (23% of requests required >3 aliquots) and lithium heparin plasma, underscoring the need for material-specific aliquoting strategies. Current broad aliquoting practices maximize flexibility but yield diminishing returns beyond two to three aliquots for most material types, at considerable cost of storage, energy, and consumables. Notably, the 6–7 year follow-up may underestimate long-term utilization: an analysis of serum samples received in 2011 showed that aliquot-level utilization increased by approximately two-thirds to ~5.5% over 14–15 years, while collection-level (50%) and submission-level utilization (21% of processed primary serum samples) remained comparable.

Prospective cohorts showed a slightly higher utilization rate (60%) than dedicated studies (48%), suggesting that hypothesis-free collection strategies may increase long-term research value by enabling broader downstream applications. This is of particular interest, as recent developments in the field of biobanking emphasize prospective on-demand collection to increase sample utilization [12,13].

Among accessed submissions, conditional median time to first request was 926 days (~2.5 years; IQR: 438–1573 days); however, Kaplan–Meier analysis demonstrated that most samples remained unused throughout follow-up, with no material reaching 50% cumulative utilization. This delay underscores the need for long-term planning in sample storage and resource allocation, particularly for hospital-based biobanks, where storage costs and energy demands are non-trivial [6,8]. The wide range of time to first use (2 to 2514 days) suggests that some samples meet immediate research needs, while others require extended storage before becoming relevant to active projects. This heterogeneity complicates cost–benefit calculations and underscores the challenge of prospective biobanking: materials are collected in anticipation of future needs that cannot be precisely predicted. The observation that 45% of collections showed no utilization within 6–7 years indicates that a substantial proportion of prospectively collected materials may never be used, or require timeframes exceeding those analyzed here. However, in absolute numbers, those 25 collections contributed only 7% of all submissions, indicating that larger (and maybe more mature) collections with an established user base are better used.

Importantly, faecal samples exhibited the shortest conditional median time to first request (162 days), yet Kaplan–Meier analysis revealed a low cumulative access rate—illustrating that rapid utilization in individual cases does not imply high overall demand. Urine and punctate samples experienced the longest delays (>1600 and 2200 days, respectively). These differences likely reflect both trends in research demand (e.g., heightened interest in microbiome analysis [14]) and logistical factors, such as ease of analysis and associated preanalytical requirements. Similarly, EDTA plasma and DNA/whole blood were accessed earlier than serum or citrate plasma, possibly because they are used in genomics and molecular assays with faster research translation timelines.

The observed near-linear access curve for serum samples contrasts with the batch-access patterns seen in several other materials, indicating serum’s ongoing demand across diverse research areas, consistent with its use in clinical chemistry, biomarker validation, and immunoassays. This is in line with Ahn et al., who reported that blood samples were the most commonly used specimens in a South Korean biobank network [15], and with a literature search by Jae-Eun et al., which identified research on plasma and serum as the most published among studies employing liquid biomaterials/matrices [16].

The observation that four material types showed zero demand over 6–7 years suggests reconsideration of their routine collection. However, the absence of recorded requests does not imply the absence of research value. First, the materials may reflect current low demand: buffy coat is increasingly bypassed in favor of direct DNA/whole-blood storage, and saliva was collected only on a small scale and from collections whose primary research focus remained on serum- or plasma-based assays. ccfDNA was requested in large quantities for samples collected at later time points. Second, the materials may be beyond the reach of affordable analytical methods, and demand may emerge as these pipelines mature. Third, the original collection strategy may itself have been suboptimal—for example, by storing only single-centrifugated EDTA plasma, which might have been used for ccfDNA isolation without accessing material dedicated for this purpose. Distinguishing these explanations will require qualitative dialog with potential users in addition to quantitative tracking and informs whether the corresponding routine pre-analytical effort should be discontinued, paused, or restructured.

A notable observation concerns the relationship between sample submitters and users. At the MedUni Wien Biobank, submitters and users are nearly always affiliated with the same clinical department. However, the biobank does not systematically track whether clinicians who request samples subsequently share them with external collaborators. Qualitative interviews conducted as part of BBMRI.at suggest that such collaborations do take place but are arranged directly between researchers rather than through the biobank. Consequently, the biobank’s potential role as a networking platform—actively facilitating cross-institutional access—may remain underutilized, even though networking at the researcher level likely takes place. This distinction has implications for how biobank “utilization” is measured and reported: current metrics capture only the initial request, not the downstream research ecosystem that may emerge from it.

The substantial heterogeneity in utilization across collections and research areas (Table 2 and Table 3) suggests that utilization is shaped by a number of factors. Without claiming a formal multivariable analysis, several plausible determinants are worth highlighting. First, the clinical area appears influential: collections supporting haematology/haemostaseology, oncology, and internal medicine show substantially higher submission-level utilization than other collections, consistent with differences in collection strategies and the maturity of biomarker pipelines in these fields. Second, sample quality and pre-analytical traceability—while uniformly documented at our biobank via SPREC and ISO/CEN-compliant procedures—may matter more for some downstream applications (e.g., proteomics, ccfDNA) than for others, possibly contributing to the limited uptake of more demanding material types. Third, the absence of a dedicated ISO 20387 accreditation, in addition to our ISO 9001:2015 certification, could have deterred some external requesters with high regulatory or industrial needs, although we are not aware of any request that was ultimately declined for this reason; pursuing ISO 20387 accreditation is currently under evaluation. It should be noted, however, that ISO 20387 was first published in August 2018, only a few months before the reported biospecimens were collected. Given that accreditation procedures typically require one to three years following standard publication, virtually no academic biobank could realistically have operated under a fully ISO 20387-accredited quality management system during the 2019 sampling period analyzed here. Lack of accreditation is therefore unlikely to have meaningfully influenced utilization within the observation window of the present study. Fourth, funding cycles plausibly synchronize batches of requests, as suggested by the stepwise rather than linear access curves observed for several material types (Figure 2). In an earlier report published by our biobank [17], the undulating access rates observed between 2010 and 2017 were interpreted as consistent with economic cycles. Fifth, awareness of biobank holdings among researchers outside the institution remains limited: although our collections are listed in the BBMRI-ERIC directory, more active outreach to the scientific community—via newsletters and scientific meetings—might be a key driver of sample uptake. Systematic capture of these determinants in future studies, including multicenter comparisons, would help disentangle their relative contributions.

These findings underscore the importance of integrating utilization data into biobank governance. Material-specific metrics on time-to-access and aliquot demands can inform evidence-based decisions regarding sample type prioritization, aliquot volume planning, and freezer capacity forecasts. The principle of “collect everything that might be needed” must be balanced against storage costs, processing effort, and environmental sustainability [18,19].

Quantitative tools such as the Biobank Economic Modeling Tool (BEMT) have been developed to support long-term financial planning based on expected utilization, highlighting the relevance of usage metrics for economic sustainability [20]. Moreover, such data enable improved alignment between biobank offerings and actual research demand, ultimately enhancing sustainability and scientific impact [7,21].

Beyond operational considerations, utilization metrics also serve as proxy indicators of biobank value. Emerging frameworks advocate for performance assessment based on sample use, scientific output, and citation of biobank resources in publications [22,23].

Looking ahead, the application of artificial intelligence and machine learning to biobank-associated data—in particular the integration of clinical, molecular, and digital pathology information, and the prediction of pathological outcomes from biobank-derived datasets—is emerging as a complementary tool for biobank research [24,25]. Such approaches may help to better match stored samples and their associated data with emerging research questions, and thereby contribute to closing the gap between collection strategy and actual research demand.

Above that, the MedUni Wien Biobank’s previous report of increasing utilization quotients from 3.5% to 6.1% between 2010 and 2017 provides additional context [17]. However, comparisons with these figures must be done with care, as they were presented as accessed aliquots relative to stored aliquots over a given time period, not as an individual sample’s utilization rate. However, as the absolute number of distributed aliquots has ranged between 7000 and 9000 samples per year since 2015 following phases of unsteady or increasing demand, it can be concluded that collections established during the biobank’s mature operational phase retain high utilization rates. This suggests that biobank performance improves with operational maturity, researcher familiarity, and refinement of collection strategies [9,26].

Several limitations must be acknowledged. First, all data originate from a single, hospital-based academic biobank. Although the MedUni Wien Biobank covers a broad spectrum of collections and a sizeable institutional research community, differences in governance, funding structure, IT infrastructure, sample portfolio, and local research culture limit the direct generalizability of our absolute utilization rates to other biobanks; the qualitative patterns we describe (the strong influence of denominator choice, the heterogeneity across material types and clinical areas, and the multi-year lag before first access) are nevertheless likely to be informative for other hospital-based biobanks and would benefit from multicenter confirmation. Second, utilization was assessed only at the level of sample access and aliquot distribution. Downstream research outputs—including peer-reviewed publications, citations of biobank resources (e.g., via CoBRA identifiers), acquired grants, and translational endpoints—were not systematically captured and are increasingly considered key performance indicators of biobank impact. Integrating such outcome metrics into biobank governance is a priority for future work, and would allow a more complete assessment of scientific return on stored material. Third, the follow-up period, while spanning up to 7 years, may not capture longer-term usage of samples with niche applications or requiring extended validation pipelines. Fourth, some materials had insufficient access events for meaningful analysis, potentially underestimating their potential utility in emerging research areas such as single-cell or cell-free nucleic acid technologies.

## 5. Conclusions

Within 6–7 years, 55% of collections were accessed, with 25.4% of submissions and 3.5% of aliquots utilized. Material-specific patterns in timing and demand provide evidence-based guidance for optimizing collection protocols and resource allocation in hospital-based biobanking. Strategically, material-specific aliquoting and storage duration policies could substantially reduce resource consumption while maintaining high request coverage, and the data invite reflection on current trends favoring on-demand biobanking.

## Figures and Tables

**Table 1:** Processing conditions of collected materials.

Biomaterial	Sample Type	Type of Primary Containers	(Usual/Aimed) Pre-Centrifugation Conditions	Centrifugation	Second Centrifugation	(Usual/Aimed) Post-Centrifugation Delay	Long-Term Storage
Serum	SER	SST, ADD, PET	A, B	B	N	D	S
Citrate plasma	PL1	SCI, PET	A	B	N	D	S
Double-spun citrate plasma	PL2	SCI, PET	A	B	B	D	S
Lithium heparin plasma	PL1	HEP, PET	A	B	N	D	S
EDTA Plasma	PL1	PED, PET	A, B	B	N	D	S
Whole blood	BLD	PED, PET	A	N	N	N	S, Y
Double-spun plasma for ccfDNA	PL2	PXD, PED, PET	A	B	B, I	D	D
Cyst punctates	ZZZ	ZZZ, PET	Ams	N	N	N	S
PAXgene RNA	BLD	PAX, PET	A	N	N	D	Y
Urine	URN	PET	A, B	A, D	N, D	C, D	S
Cerebrospinal fluid	CSF	PET, PPS	A	B	N	B	S
Faeces	STL	ZZZ, PPS	A	N	N	N	B, D
Saliva	SAL	ORG, PPS	A	B, N	N	D	S

SPREC v2.0 codes are given for the materials collected in 2019 [11].

**Table 2:** Collection-specific metrics.

Collection	Collection Type	Submissions		Primary Materials		Aliquots	
		Received	Accessed	Received	Accessed	Received	Distributed
1	C	50–100	<10 (<10%)	300–500	<10 (<10%)	1000–2000	<10 (<10%)
2	C	50–100	<10 (<10%)	200–300	<10 (<10%)	1000–2000	<10 (<10%)
3	C	300–500	<10 (<10%)	500–1000	<10 (<10%)	1000–2000	<10 (<10%)
4	C	1000–2000	50–100 (<10%)	3000–5000	50–100 (<10%)	10,000–100,000	50–100 (<10%)
5	C	3000–5000	200–300 (<10%)	>10,000	200–300 (<10%)	10,000–100,000	200–300 (<10%)
6	C	50–100	10–20 (10–20%)	200–300	10–20 (<10%)	1000–2000	10–20 (<10%)
7	C	1000–2000	200–300 (20–30%)	3000–5000	300–500 (<10%)	10,000–100,000	300–500 (<10%)
8	C	50–100	10–20 (20–30%)	300–500	10–20 (<10%)	1000–2000	10–20 (<10%)
9	S	100–200	30–50 (20–30%)	100–200	30–50 (20–30%)	300–500	30–50 (<10%)
10	C	3000–5000	500–1000 (20–30%)	>10,000	500–1000 (<10%)	10,000–100,000	500–1000 (<10%)
11	C	100–200	50–100 (30–40%)	500–1000	50–100 (10–20%)	2000–3000	100–200 (<10%)
12	S	300–500	200–300 (40–50%)	500–1000	200–300 (20–30%)	3000–5000	200–300 (<10%)
13	C	<50	10–20 (40–50%)	300–500	10–20 (<10%)	500–1000	50–100 (10–20%)
14	C	500–1000	300–500 (50–60%)	2000–3000	500–1000 (20–30%)	10,000–100,000	500–1000 (<10%)
15	S	<50	10–20 (50–60%)	<50	10–20 (60–70%)	300–500	10–20 (<10%)
16	C	<50	10–20 (50–60%)	50–100	10–20 (20–30%)	500–1000	20–30 (<10%)
17	C	500–1000	300–500 (60–70%)	1000–2000	500–1000 (30–40%)	5000–10,000	1000–1500 (10–20%)
18	C	50–100	30–50 (60–70%)	100–200	30–50 (30–40%)	500–1000	30–50 (<10%)
19	S	100–200	50–100 (70–80%)	300–500	100–200 (40–50%)	1000–2000	300–500 (20–30%)
20	C	50–100	50–100 (80–90%)	200–300	50–100 (40–50%)	1000–2000	300–500 (30–40%)
21	C	100–200	100–200 (90–100%)	300–500	100–200 (40–50%)	2000–3000	300–500 (10–20%)
22	S	<50	10–20 (90–100%)	<50	10–20 (90–100%)	100–200	30–50 (30–40%)
23	C	50–100	30–50 (90–100%)	100–200	50–100 (50–60%)	500–1000	200–300 (20–30%)
24	C	200–300	200–300 (90–100%)	500–1000	500–1000 (60–70%)	5000–10,000	1000–1500 (10–20%)
25	S	<50	20–30 (90–100%)	50–100	30–40 (60–70%)	200–300	50–100 (30–40%)
26	S	50–100	50–100 (90–100%)	100–200	50–100 (40–50%)	300–500	50–100 (10–20%)
27	C	<50	<10 (90–100%)	<50	10–20 (80–90%)	100–200	50–100 (40–50%)
28	C	<50	10–20 (90–100%)	<50	10–20 (40–50%)	100–200	10–20 (<10%)
29	S	<50	<10 (90–100%)	<50	<10 (90–100%)	<50	10–20 (70–80%)
30	S	<50	<10 (90–100%)	<50	<10 (20–30%)	300–500	30–50 (<10%)
31	S	<50	20–30 (90–100%)	<50	30–50 (90–100%)	100–200	100–200 (90–100%)
Total (56 collections)		13,666	3466 (25.4%)	46,288	4537 (9.8%)	218,257	7565 (3.5%)
Total (31 utilized)		12,693	3466 (27.3%)	44,276	4537 (10.2%)	207,741	7565 (3.6%)

C, hypothesis-free cohort; S, specific study. Numbers are presented as ranges to protect collection confidentiality.

**Table 3:** Utilization according to research area.

Research Area	Utilized Submissions	Utilized Aliquots
Autoimmunity	16%	2%
Cardiology	54%	14%
Haematology/Haemostaseology	85%	26%
Oncology	71%	17%
Transplantation	13%	1%
Other (Internal medicine)	81%	12%
Other	30%	2%
Total	27.3%	3.6%

Categories containing fewer than three different collections are summarized as “Other (internal medicine)” or “Other” respectively.

**Table 4:** Time to first use per material type among accessed samples.

	Serum	Cit	LiHep	EDTA	DNA/WB	Urine	Faeces	CSF	Pax	Punct	All Materials
Mean	908	1076	1135	725	645	1394	529	620	987	2182	1011
SD	606	694	28	419	576	552	448	371	85	122	634
Median	808	767	1133	578	312	1604	162	446	1021	2233	926
Q1	428	616	1124	384	216	1222	137	343	920	2152	438
Q3	1292	1934	1158	1040	1256	1796	1009	930	1053	2250	1573
Min	2	4	1082	10	48	185	118	77	783	1959	2
Max	2514	2204	1166	2135	2255	1856	1048	1853	1105	2288	2514

Figures represent the number of days from reception at the biobank to the first request by a biobank user. Colors rank material types from fast (green) to slow (red) utilization. SD, standard deviation; Q1, 1st quartile; Q3, 3rd quartile; Min, shortest time; Max, longest time; Cit, citrate plasma; LiHep, lithium heparin plasma; EDTA, EDTA plasma; DNA/WB, DNA or EDTA-anticoagulated whole blood for DNA isolation; CSF, cerebrospinal fluid; Pax, PAXgene; Punct, cyst punctate.

**Table 5:** Aliquot requirements per material type.

	Serum	Cit	LiHep	EDTA	DNA/WB	Urine	Faeces	CSF	Pax	Punct
N	1733	668	12	494	356	1141	18	77	32	6
Mean	1.7	2.6	4.9	1.8	1.1	1.2	1	1.6	1.0	1
SD	1.2	2.0	0.3	1.4	0.3	0.4	0	0.8	0.2	0
Median	1	2	5	1	1	1	1	1	1	1
Q1	1	1	5	1	1	1	1	1	1	1
Q3	2	3	5	2	1	1	1	2	1	1
Minimum requested aliquots per sample	1	1	4	1	1	1	1	1	1	1
Maximum requested aliquots per sample	9	10	5	6	4	3	1	5	2	1

N, total amount of accessed samples (at least 1 aliquot) received in 2019; SD, standard deviation; Q1, 1st quartile; Q3, 3rd quartile; Min, minimum distributed aliquots per sample; Max, maximum distributed aliquots per sample; Cit, citrate plasma; LiHep, lithium heparin plasma; EDTA, EDTA plasma; DNA/WB, DNA or EDTA-anticoagulated whole blood for DNA isolation; CSF, cerebrospinal fluid; Pax, PAXgene; Punct, cyst punctate.

## Data Availability

The data presented in this study are included in the article (in an aggregated form); more detailed data are available on request from the corresponding author due to data protection reasons.

## Abbreviations

The following abbreviations are used in this manuscript:

BBMRI-ERIC	Biobanking and BioMolecular Resources Research Infrastructure—European Research Infrastructure Consortium
BEMT	Biobank Economic Modeling Tool
ccfDNA	cell-free circulating DNA
CEN	European Committee for Standardization
Cit	citrate plasma
CSF	cerebrospinal fluid
DNA/WB	DNA or EDTA-anticoagulated whole blood for DNA isolation
EDTA	ethylenediaminetetraacetic acid
IQR	interquartile range
ISO	International Organization for Standardization
LiHep	lithium heparin plasma
Max	maximum
Min	minimum
MOLIS	laboratory information management system (vision4health)
N	number
Pax	PAXgene RNA
Punct	cyst punctate
Q1	first quartile
Q3	third quartile
SD	standard deviation
SPREC	Standard PREanalytical Code
